# Sustainable Tourism as a Source of Healthy Tourism

**DOI:** 10.3390/ijerph17155353

**Published:** 2020-07-24

**Authors:** Luna Santos-Roldán, Ana Mª Castillo Canalejo, Juan Manuel Berbel-Pineda, Beatriz Palacios-Florencio

**Affiliations:** 1 Department of Statistics, Business Organization and Applied Economics, Faculty of Law and Business, University of Córdoba, 14071 Córdoba, Spain; acastillo@uco.es; 2 Department of Business Organization and Marketing Faculty of Business Studies, University of Pablo de Olavide, CarreteraUtrera, Km.1, 41013 Seville, Spain; jmberpin@upo.es (J.M.B.-P.); bpalacios@upo.es (B.P.-F.)

**Keywords:** sustainable tourism, attitude, positive effects, motivation, satisfaction

## Abstract

Even though the World Tourism Organization described Sustainable Tourism as a tourism form that could contribute to the future survival of the industry, the current reality is quite different, since it has not been firmly established in society at expected levels. The present study analyzes which variables drive the consumption of this tourism type, taking tourist awareness as the key element. To this awareness, we must add the current crisis experienced by the tourism industry caused by COVID-19, since it can benefit Sustainable Tourism development, promoting less crowded destinations that favor social distancing. For this, the existing literature on Sustainable Tourism has been examined in order to create a model that highlights the relations among these variables. To determine the meaning of these relations, a sample of 308 tourists was analyzed through structural equation models using Partial Least Squares. The results show that there is a clear attitude on the part of the tourist to develop Sustainable Tourism, driven by the positive effects and motivation it entails, as well as the satisfaction the tourist perceives when consuming a responsible tourism type.

## 1. Introduction

The World Tourism Organization (UNWTO) in 2005 defined the concept of Sustainable Tourism as “one whose practices and principles can be applicable to all forms of tourism in all types of destinations, including mass tourism and the various niche tourism segments”. Sustainability principles refer to the environmental, economic, and socio-cultural aspects of tourism development, and a suitable balance must be established among these three dimensions to guarantee its long-term sustainability [1]. In addition to international organizations, we also find many authors who have defined the concept of sustainable tourism [2,3,4,5,6]. Conversely, despite the fact that sustainable tourism has been recognized in business practice, the volume of academic research has not been as relevant as might be expected [7]. From the start, the development of sustainable tourism is based on environmental preservation, cultural authenticity and the profitability of the tourist activity in the destination [8]. In this tourism type, both social return and the reversed well-being index on the visited destinations are recognized, as well as the economic return—in other words, whether the tourist activity generates enough income for the local population in terms of employment, wealth and available resources [9].

This study aims to establish the factors that allow sustainable tourism development, which is really necessary for the industry in the context of the crisis caused by COVID-19. From a theoretical point of view, motivational factors, economic impact and satisfaction are analyzed as attributes that potentially influence the intention and attitude of choosing this type of tourism. 

Hence, one of the possible solutions the tourism industry can find to help the current crisis that it faces as a consequence of COVID-19 could come from Sustainable Tourism. Finding solutions is more than necessary in those countries where the tourism industry plays an important role in the economy. Thus, in the case of Spain, it must be considered that it is a country highly dependent on tourism. In 2019, it was the second world destination in terms of international tourist arrivals (83.3 millions), with EUR 92.5 billion in tourism revenue, 2.8 million direct jobs and a contribution to GDP of 14.2% [10]. In this way, tourism is considered the main industry in the country. Therefore, in order to preserve this situation, it is essential to promote developing a sustainable industry over time and, perhaps more necessary than ever, this tourism type.

The contribution of this research is double-edged. Firstly, we present a model that relates a set of variables obtained from the literature and that must be considered for sustainable tourism development from the perspective of the tourist (applicant for tourist services). Secondly, we propose a hypothesis set that seeks to analyze both the level and strength of these relations as drivers of an attitude favorable to sustainable tourism development. The study begins with a review of the literature to consider the relation among the variables considered in the study. Next, the methodology used in the data collection is explained to later expose the results analysis, as well as the discussion and conclusions, which complete the final sections of this study.

## 2. Literature Review

### 2.1. Relation between Positive Impacts and Attitude towards Sustainable Tourism

According to [11], tourism impacts are the result of human behavior stemming from interactions between tourists and the subsystems of the territory where they come into play. Throughout the publications that take the study of sustainable tourism as a main topic, the doctrine that corroborates the effects of positive impacts is predominant, whose consequences affect residents, economy and environment [12]. Firstly, the main positive economic aspects are based on greater economic movement, contribution to GDP, job creation and income distribution in other local economic activities. Secondly, regarding residents, the well-being of the local and tourist population is taken into account meticulously, in addition to the respect and preservation of the culture and heritage of the host region. Thirdly, as sustainable tourism is closely related to the environment, due to its use of natural resources, it highly depends on having an attractive natural environment. This produces an increased environmental awareness in society, as well as the revaluation of the natural environment through the approval of environmental quality conservation, protection and improvement measures [13,14,15,16].

Sustainable tourism development is inherent to those tourists capable of showing a greater awareness of the sustainability problem [17], who are averse to mass tourism development and seek to contribute to destination protection when choosing. Sensitive to the negative impacts of tourism, they support the development of respectful sustainable tourism from an economic, social and environmental perspective [18,19]. In accordance with [20], that the positive impacts of awareness of the protection of natural resources, cultural resources and the increase in local recreational facilities and resources were considered. From the study of these authors, the following hypothesis is proposed:

**Hypothesis** **1 (H1).** *The positive impact on tourists has a direct and positive influence on their attitude towards sustainable tourism development.*

### 2.2. Relation between Satisfactory Experience and Attitude towards Sustainable Tourism

Customer motivation is identified as a determinant factor in the success of all industries [21] and, homogeneously, in the case of the tourism sector, influences future intentions of purchase and visiting of the same destination [22]. The success of a global model of sustainable tourism requires achieving high levels of tourist satisfaction, thus increasing their awareness of the problems that sustainability encompasses and promoting more respectful practices. This long-term maintenance of the applicant’s satisfaction guarantees the consolidation of the destination in the market and, at the same time, it favors an adequate demand according to its attractions [23]. This satisfaction is configured based on previous expectations and evaluation after finishing the tourist experience [24,25]. 

Recent studies have analyzed this direct relation between both constructs, linked to a given geographic environment [25,26,27,28]. This study covers a geographic area not detected in the literature review and, in line with the proposed authors, the second of the hypotheses is established:

**Hypothesis** **2 (H2).** *Experiential satisfaction has a direct and positive influence on the attitude towards sustainable tourism.*

### 2.3. Motivation and Attitude Relation towards Sustainable Tourism

Motivation has been analyzed as an internal factor that guides and integrates the behavior of the individual. It is a psychological factor that leads people to act in a certain way to satisfy their desires and goals [29] and, therefore, a driver that motivates people to take vacations or visit destinations [4]. Motivation is related to the attitudes and intentions of tourists when choosing a destination [30,31], and the experience gained in situ is crucial to satisfy that motivation and increase the loyalty to a tourist destination [32]. Hence, tourist motivation is not only useful to explain tourist behavior, but also acts as a predictor of the visit intention [33].

The customer’s profile of sustainable tourism involves a tourist committed to the environment and who is aware of sustainability. As tourist motivation has positive effects on the visit intention, various studies [34,35,36,37] confirm that the experience is more attractive to tourists when they participate in activities entailing more responsible behavior and greater involvement with the environment, the local community and society. In this way, a direct relation between motivation and attitude towards sustainable tourism is established. From the reading of these authors, the third hypothesis is proposed:

**Hypothesis** **3 (H3).** *Motivation has a direct and positive influence on the attitude towards sustainable tourism.*

### 2.4. Moderating Effect of Motivation in the Relation between Positive Impacts and Attitude towards Sustainable Tourism

Most research concludes that the three basic categories of benefits and costs that affect a community which receives tourists are economic, environmental, and social [5,38,39,40,41,42,43], although [2] also incorporate institutional sustainability. Likewise, the sustainability principles imply a balance of these three dimensions: environment, economy and society [32]. Most studies report a positive relation between the attitude towards sustainable tourism development and the perception of its positive impacts [2,5,26,44,45,46].

In the tourism context, motivation is one of the most important values regarding behavioral intentions among revisiting a place, word of mouth and the search for alternative destinations [47]. This is why tourists more committed to the balance among sustainability dimensions show a higher motivation towards this tourism type and, on the other hand, there is a direct and positive relation between these two variables, as other authors have analyzed [36,37]. Therefore, research has focused on the direct relation between positive impacts and motivation with an implication towards sustainable tourism (hypotheses 1 and 3 of the model); however, the moderating effect that motivation can have on positive impacts and sustainable tourism has not been analyzed. Therefore, the following hypothesis is formulated:

**Hypothesis** **4 (H4).** *Motivation has a moderating effect on positive impacts and the attitude towards sustainable tourism.*

### 2.5. Moderating Effect of Motivation in the Relation between Satisfaction and Attitude towards Sustainable Tourism

Understanding what factors influence tourist satisfaction is one of the most relevant research topics in the tourism sector, due to the impact it has on the success of any tourism product or service. Most tourists can compare the aspects of different destinations (such as services, attractions, etc.) according to their perceptions. A high level of tourist satisfaction fosters positive future behaviors, such as the intention to revisit and recommend a destination [48].

The relation of satisfaction and motivation with the attitude towards sustainable tourism has been analyzed in the scientific literature by various studies [36,37,49,50,51] to refer to the tourists’ general evaluations of their experiences with environment respect and their expectations regarding the sustainable development of tourist destinations or services. Similarly, the moderating effect of satisfaction with sustainable tourism and other variables, such as the recommendation of a destination and emotional value, have also been analyzed [32]. However, the moderating effect that motivation can have on the relation between satisfaction and attitude towards sustainable tourism has not been examined. Consequently, we deduce from the collected studies that the higher the motivation of a tourist to visit ecological and sustainable environments is, the greater the impact of said relation is. Therefore, the following model hypothesis is suggested:

**Hypothesis** **5 (H5).** *Motivation has a moderating effect between satisfaction and attitude towards sustainable tourism.*

**Figure 1:**
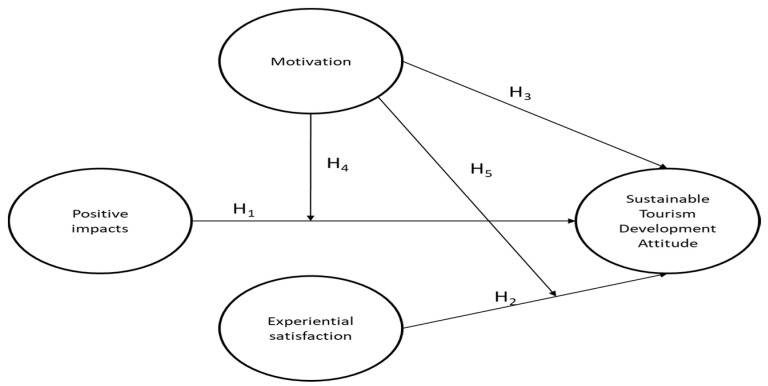
Conceptual model. Source: Own elaboration. While H1, H2 and H3 consider a direct influence between constructs, H4 and H5 include a moderator perspective.

All hypothesis are represented in Figure 1:

## 3. Data and Methodology

### 3.1. Participants

The data were collected from tourists who visited the city of Córdoba (Spain) between the months of October and November 2019. Córdoba (a World Heritage city) is one of the main cities receiving tourism (both national and international). This means that one of the main challenges it faces is its massification and the problems associated. The measurement instrument was completed by a total of 308 subjects.

Table 1 shows the main aspects related to the respondents’ profiles. It has to be emphasized that a relative equality is observed regarding the origin of the tourists surveyed. In general, these are tourists who came on holidays that they financed and, in a high percentage of cases, that were based on their own decisions. The questionnaire was personally distributed among the main tourist attractions located inside the old quarter. More than 60% of the travelers were 35 years old or more, had a stable partner (married and living as a couple), lived in households based on two or more people and earned a monthly income of between EUR 1000 and 2000. Although the majority of the respondents stated that they were visiting this city for the first time (62.7%), a high percentage (close to 40%) repeated their destination. On average, the tourists who participated in the study stayed in the city for 2–3 days.

### 3.2. Measurements 

In relation to the instruments used, special interest was placed on translating the original versions of the scales to the linguistic characteristics of the population. All variables were measured on a Likert scale from 1 to 5, where 1 = totally disagree and 5 = totally agree. 

The items in the questionnaire were translated and adapted for the different constructs. The items of positive socio-cultural, economic and environmental impacts were extracted and adapted from [20]. The items related to experiential satisfaction were obtained from the research carried out by [51]. The items of Sustainable Tourism Development Attitude were adapted from the study of [26]. Finally, the construct of motivation was extracted and adapted from [4].

### 3.3. Data Analysis

The relations among the variables, with special emphasis on the moderating effect of the satisfaction experience, were analyzed with a structural equation model based on variance—the Partial Least Squares (PLS). Furthermore, the recommendations of various authors were followed: [52] and [53]. The computer software used was SmartPLS 3.2.8.

The use of a single instrument to collect data on constructs (latent variables) implies the need to check the existence of a common variance among them. Following the experts’ opinions [54,55] on the design and execution process of questionnaires, we proceeded to separate the different measures, as well as to guarantee the anonymity of the respondents. The presence of common influence on responses was analyzed using the [56]. The exploratory factor analysis recorded the existence of 12 factors, where the largest of them explains 17% of the total variance. Therefore, there is no common factor of influence among the items included in the questionnaire [57].

The validation of the proposed model was carried out at a double level. Firstly, the measurement model. Once the validity of the model was checked, the structural model was validated.

## 4. Results and Discussion

### 4.1. Measurement Model

The mean and standard deviation values of each item, as well as the latent variables, are given in Table 2. In addition, this table includes the data required to perform the first step in the validation of the measurement model: determining the reliability of the individual items. It can be seen that the factorial loads of most of the items exceed the minimum criterion of 0.707 [58]. Only one item with a lower value has been maintained, although it is very close (0.692). This item was not eliminated after checking its significance level via bootstrapping (5000 subsamples) and in accordance with the suggestions of [59]. Finally, 22 elements of a total of 31 items related to the constructs of the model analyzed were removed.

In order to set the construct’s reliability, the composite reliability index (ρ_c_) was used [60]. All its values, included in Table 2, are above the minimum threshold: above 0.7 [61]. The previous table also shows the AVE value, which is used to determine the convergent validity, since it exceeds the minimum level of 0.5 in all latent variables [62].

To determine the discriminant validity of the model constructs, we used the Fornell–Larcker test and the corresponding values are shown in Table 3. These table data verify that all the constructs strictly meet the Fornell–Larcker criterion. In short, this allows us to affirm the existence of discriminant validity among the latent variables and the way of measuring them. 

After validating the measurement model, it is necessary to validate the structural model.

### 4.2. Structural Model

Following the opinion of [63], this study should start with the analysis of the sign, size and significance of the path coefficients, the values of R^2^ and the Q^2^ test. [59] recommend the use of the bootstrapping technique with 5000 samples to calculate the t statistics and the confidence intervals, which will allow for establishing the significance of the relations. The two-step technique has been followed for the analysis of the moderating effects [59]. Table 4 shows the direct effects (path coefficients), the values of the t statistic, the corresponding confidence intervals (without bias) and the verification of whether the proposed hypotheses have been supported, without forgetting the values of R^2^ and Q^2^. 

In order to have a greater awareness of the attitude of the tourist (consumer of tourist services) towards sustainable tourism development, it is necessary to know what variables influence it. Based on previous work on Sustainable Tourism, this study designs a model where a set of variables that influence the attitude towards the development of Sustainable Tourism are described. The relations among them and the consistency of this relation have also been analyzed.

**Figure 2:**
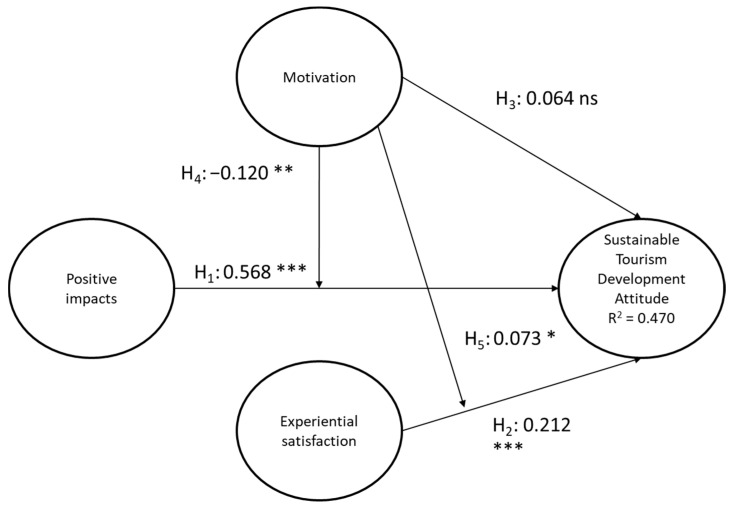
Structural model.

Not all the hypotheses presented have been validated. The hypothesis that relates positive economic impacts to attitude presents higher parameters (β, T-Student) than other variables (GIP ATT; β = 0.568; *t* = 14.393). Furthermore, the relation with satisfaction for the experience had developing this modality of tourism shows quite adequate levels (SAT ATT; β = 0.212; *t* = 4.957). This reveals the important role played by the tourist’s awareness and their contribution so that Sustainable Tourism can be developed. Lower levels are found in the hypothesis that relates motivation to attitude (MOT ATT; β = 0.064; *t* = 1.438). This means that this relation is presented as “not significant”. However, the variable “motivation” also has a moderating effect on the relations shown in the model, both the one we observe in positive impacts and the one that appears in satisfaction on the attitude for sustainable tourism development. Thus, we find that, as motivation increases, the satisfaction effect on attitude also grows (β = 0.073; *t* = 1.852). However, the moderating effect of the variable “motivation” is the opposite in the relation between positive impacts and attitude (β = −0.120; *t* = 2.640). In other words, the less motivation there is, the less the impact effect on the attitude will be reduced (Figure 2).

This study is in line with previous works that have highlighted the importance of tourist awareness for sustainable tourism development [12,24,25,28,37]. Similarly, it is in line with the recently published document “Covid-19: EU Guide to the progressive resumption of tourism services and health protocols in hotels” [64]. It is essential to develop Covid-19-related protocols for hotel establishments [65,66,67]. These protocols corroborate many of the guidelines that must be followed for sustainable tourism development, fundamentally related to environmental protection, based on a commitment to reduce destination massification in order to avoid tourist overcrowds. In this way, it will be possible to maintain a safe distance among people while consuming tourist services, which comprises the main concern of the health authorities. Nevertheless, we note that the health authorities have not clearly communicated these protocols yet. This lack of information will lead to a certain delay in the implementation of measures in tourist offers, which could help sustainable tourism development. 

We believe that, as health authorities establish the protocols to responsibly and safely develop tourism, and as long as the tourists are aware of their implementation, it is quite probable that the level of the different variables of the proposed model (positive impacts, satisfaction and motivation towards the attitude for sustainable tourism development) will increase due to a greater knowledge on the part of the tourist. A higher level of awareness, fundamentally supported by the security that tourists need in the current context, can lead them to consume tourist services.

## 5. Conclusions

The development of sustainable tourism requires, mainly, an awareness on the part of the tourist services applicant (the tourist). According to the analysis of the literature, we find a series of factors that can contribute to increasing positive attitudes towards sustainable tourism development from the perspective of demand. Through this analysis and a methodology focused on personal surveys carried out on tourists using a questionnaire, the factors that make tourists have a favorable attitude towards sustainable tourism development have been determined. Based on this study and the results obtained from the fieldwork, in addition to the analysis of the tourist-industry situation after the health crisis, a series of conclusions are drawn from both theoretical and empirical perspectives.

### 5.1. Theoretical Perspective

Firstly, in the literature, we find three factors that lead to a favorable attitude for sustainable tourism development from the tourist’s perspective. On the one hand are the positive effects perceived by the tourist with the consumption of sustainable tourism, whose consequences affect the resident’s, economy and destination environment [12,16]. On the other hand, customer satisfaction is identified as a determinant of success in any industry [21] and, therefore, in the tourism industry [28]. Finally, motivation is related to the attitudes and intentions of tourists when choosing a destination [30,31]. However, this motivation plays a moderating (and different) role in the relation between the positive effects and the attitude towards sustainable tourism development and in the relation between satisfaction and the attitude towards sustainable tourism. Thus, motivation harms the positive impact and favors the satisfaction effects on the attitude of sustainable tourism development.

Secondly, after reviewing studies and reports on the current context of the tourism industry, it can be affirmed that there is a need to develop protocols (based on the perspective that tourist services offer) which reaffirm tourists’ commitments to consume sustainable tourism. This perspective is not included in the model (which only analyzes the demand perspective); however, from a theoretical point of view, it does seem necessary to consider. In this scenario, the performance of the presented empirical study is one more reference to contemplate that the tourist (applicant for tourist services) becomes a fundamental element for sustainable tourism development. Nevertheless, for their safety, the tourist will demand tourist services that take into account hygiene and health factors [65] and aspects related to social distancing [68,69].

### 5.2. Empirical Perspective

Firstly, only two of the three main hypotheses regarding the direct effects are supported. The attitude towards sustainable tourism development is positively influenced by the global positive impacts perceived by the tourist and by the satisfaction experienced with the consumption of this tourism type. The value of R^2^ indicates an appropriate predictive level for the “attitude towards sustainable tourism development” variable, which is reinforced by the value of Q^2^.

Secondly, it is important to highlight that the “motivation” variable plays a moderating role regarding the impacts on the relations of the other two variables analyzed. In the case of positive global impacts, the incidence of motivation is negative—in other words, the less motivated a subject is, the lower the effect of global positive impacts on the attitude towards sustainable tourism development will be. On the other hand, motivation seems to increase the effect of the satisfaction experienced on the attitude towards sustainable tourism development.

In summary, sustainable tourism can build on the momentum provided by this context of health crisis to increase a favorable attitude towards sustainable tourism development. Achieving this attitude will allow for an increase in the tourist’s awareness, translated as the tourist’s perception of the positive impact and the satisfaction experienced with the consumption of this tourism type. Likewise, motivation is a factor that also favors the attitude towards sustainable tourism development. At the same time, a priori, the role played by the tourist offer (companies and authorities) to help increase this tourist awareness will also be important.

### 5.3. Limitations and Future Studies

The main limitation we find in the present work is that the study was carried out prior to the crisis caused by COVID-19; therefore, aspects such as changes in attitudes, motivations and perceptions related to and caused by this new situation were not considered at any time. However, the study focused on analyzing the importance of sustainable tourism. Without doubt, this relevance will be driven by this new situation. Therefore, it would be very convenient to replicate this work in the future when total mobility within national territories begins to be allowed and the circulation of tourists is allowed internationally. Thus, for example, an aspect that was not supported in the present work is the importance of positive impacts a destination could have, which did not suppose a motivation for its choice. Thanks to the different government campaigns that are being recently launched with the aim of achieving positive impacts from visiting certain destinations, we think that this aspect could change due to the increased sensitivity perceived by the tourist. In short, it is necessary to find formulas that boost the tourism industry. The development of sustainable tourism can help to mitigate tourists’ perceived fears of visiting destinations with a large concentration of people.

Therefore, it would also be interesting to launch a study that considers the actions carried out by the tourism offer in order to verify its impact on the attitude towards sustainable tourism development.

## Figures and Tables

**Table 1:** Respondent characteristics.

Descriptive Variables	Absolute Frequency	Percentage
**Sex**		
Male	142	46.1
Female	166	53.9
**Nationality**		
Spaniard	163	52.9
Other	145	47.1
**Visit purpose**		
Work	23	7.5
Vacancy	150	48.7
Visit friends	47	15.3
Family event	25	8.1
Independent journey	39	12.7
Others	24	7.8
**Person who paid for the visit**		
Myself	140	45.5
My company	29	9.4
My partner	63	20.5
Friends	10	3.2
Family	60	19.5
Others	6	1.9
**Person who proposed the destination**		
Myself	111	36.0
My company	31	10.1
My partner	55	17.9
Friends	36	11.7
Family	71	23.1
Others	4	1.3
**Professional status**		
Student	54	17.5
Freelance	43	14.0
Employed person	141	45.8
Unemployed person	16	5.2
Retired person	19	6.2
Homemaker	28	9.1
Lost	7	2.3
**Age**		
65 years old or more	21	6.8
55–64 years old	45	14.6
45–54 years old	79	25.6
35–44 years old	57	18.5
26–34 years old	43	14.0
18–25 years old	62	20.1
Lost	1	0.3
**Marital status**		
Single	78	25.3
Married	135	43.8
Common-law relationship	60	19.5
Divorced	27	8.8
Widow/er	7	2.3
Lost	1	0.3
**Household size**		
Individual	37	12.0
2 people	126	40.9
3 people	73	23.1
4 people	58	18.8
5 or more people	14	4.5
Lost	2	0.6
**Salary**		
Less than EUR 999	71	23.1
EUR 1000–1499	96	31.2
EUR 1500−1999	72	23.4
EUR 2000 and over	60	19.5
Lost	9	2.9

Source: Own elaboration.

**Table 2:** Mean, standard deviation, individual reliability, composed reliability and average variance extracted for constructs and indicators.

Construct and Indicator	Mean	SD	Loading	Composed Reliability	AVE
Global positive impacts (GPI)	3.35	1.110		0.885	0.719
Tourism promotes awareness of the protection of natural resources	3.13	1.237	0.807		
Tourism promotes awareness of the protection of cultural resources	3.44	1.324	0.877		
Tourism increases local recreational facilities and resources	3.46	1.366	0.858		
Sustainable Tourism Development Attitude (SUS)	3.43	0.991		0.800	0.668
I think the attitudes and behaviors of local tourists are satisfactory and do not disturb residents	3.25	1.106	0.743		
I think that the positive aspects of the development of Sustainable Tourism are greater than their negative aspects	3.61	1.306	0.885		
Experiential satisfaction (SAT)	4.02	0.803		0.765	0.622
It is worth visiting a sustainable city	4.07	1.062	0.692		
I feel that I contribute to environment protection and Sustainable Tourism	3.97	0.941	0.875		
Motivation (MOT)	4.46	0.585		0.749	0.600
I want to travel somewhere that offers an ecological environment	4.17	0.900	0.825		
I want to experience different cultures from mine	4.75	0.574	0.720		

Source: Own elaboration.

**Table 3:** Constructs discriminant validity (Fornell–Larcker criterion).

Constructs	GIP	MOT	SUS	SAT
GIP	**0.848**			
MOT	0.190	**0.774**		
SUS	0.646	0.215	**0.817**	
SAT	0.303	0.131	0.382	**0.789**

GIP: Global positive impacts; MOT: Motivation; SUS: Sustainable Tourism Development Attitude; SAT: Experiential satisfaction. Diagonal elements (bold figures) are the square root of the variance shared between the constructs and their measures. Off-diagonal elements are the correlations among constructs. For discriminant validity, diagonal elements should be larger than off-diagonal.

**Table 4:** Direct effects on endogenous variables.

Effects on Endogenous Variables	Path (β)	*t* Value (Bootstrap)	Confidence Interval	Explained Variance	Support
**Sustainable Tourism Development Attitude** Adj R^2^ = 0.470/Q^2^ = 0.297)					
H1: Global positive impacts	0.568 ***	14.393	(0.500; 0.627) Sig	36.69%	Yes
H2: Experiential satisfaction	0.212 ***	4.957	(0.137; 0.279) Sig	8.09%	Yes
H3: Motivation	0.064 ns	1.438	(−0.015; 0.133)	1.38%	No
H4: Global positive impacts x Motivation (interaction term)	−0.120 **	2.640	(−0.195; −0.046) Sig		Yes
H5: Experiential satisfaction x Motivation (interaction term)	0.073 *	1.872	(0.012; 0.140) Sig		Yes

*** *p* < 0.001, ** *p* < 0.01, * *p* < 0.05; ns: not significant.; nd: not determined. *t* (0.05; 4999) = 1.645, *t* (0.01; 4999) = 2.327, *t* (0.001; 4999) = 3.092. One-tailed test.

## References

[1] (2005). UNWTO. https://www.unwto.org/sustainable-development.

[2] Hussain K., Ali F., Ari Ragavan N., Singh Manhas P. (2015). Sustainable tourism and resident satisfaction at Jammu and Kashmir, India. Worldw. Hosp. Tour. Themes.

[3] Zamfir A., Corbos R.A. (2015). Towards Sustainable Tourism Development in Urban Areas: Case Study on Bucharest as Tourist Destination. Sustainability.

[4] Mohaidin Z., Tze Wei K., Ali Murshi M. (2017). Factors influencing the tourists’ intention to select sustainable tourism destination: A case study of Penang, Malaysia. Int. J. Tour. Cities.

[5] Chi-Ming H., Chang H., Sung Hee P. (2017). A study of two stakeholders’ attitudes toward sustainable tourism development: A comparison model of Penghu Island in Taiwan. Pac. J. Bus. Res..

[6] Higgins-Desbiolles F., Carnicelli S., Krolikowski C., Wijesinghe G., Boluk K. (2019). Degrowing tourism: Rethinking tourism. J. Sust. Tour..

[7] Ruhanen L., Weiler B., Moyle B.D., McLennan C.L.J. (2015). Trends and patterns in sustainable tourism research: A 25-year bibliometric analysis. J. Sust. Tour..

[8] Crosby A. (1996). Elementos Básicos para un Turismo Sostenible en las áreas Naturales.

[9] Fernández Poncela A.M. Turismo, Negocio o Desarrollo: El Caso de Huasca, México. https://riull.ull.es/xmlui/handle/915/16470.

[10] (2020). INE. https://www.ine.es/.

[11] Ardahaey F.T. (2011). Economic impacts of tourism industry. Int. J. Bus. Manag..

[12] Abdallah A., Al-Bakry R. (2019). Advancing Towards Sustainable Economies: Examining Resident Attitudes and Perceptions Towards Sustainable Tourism Development in Qatar.

[13] Fullana P., Ayuso S. (2002). Turismo Sostenible.

[14] Meuser T., Von Peinen C. Sustainable Tourism “Wish you Weren’t Here”. https://link.springer.com/chapter/10.1007/978-3-8349-7043-5_5.

[15] Cardona J.R., Azpelicueta Criado M.C., Serra Cantallops A. (2015). Proposal for general components of residents’ attitudes: Traditional society, development of tourism and evolution of attitudes. Revista Brasileira de Pesquisa em Turismo.

[16] Santiago Escobar D.M. (2018). Turismo Sostenible y Desarrollo: Análisis del Desarrollo Turístico Sostenible Colombiano Mediante el Estudio de la Efectividad de Los Programas de Asistencia al Desarrollo Como Modelos de Ayuda a la Sostenibilidad Local.

[17] Rivas García J., Díaz Magadán M. (2007). Los indicadores de sostenibilidad en el turismo. Rev. Econ. Soc. Tur. Medio Ambiente.

[18] Cottrell S., Duim R., Ankersmid P., Kelder L. (2004). Measuring the sustainability of tourism in Manuel Antonio and Texel: A tourist perspective. J. Sustain. Tour..

[19] Ohtman N., Anwar N.A.M., Kian L.L. (2010). Sustainability analysis: Visitors’ impact on Taman Negara. J. Tour. Hosp. Culin. Arts.

[20] Pavlić I., Portolan A., Puh B. (2017). (Un)supported current tourism development in UNESCO protected site: The case of old city of dubrovnik. Economies.

[21] Adetola B.O., Adewumi I.B., Olonimoyo H.T. (2016). Tourist Satisfaction with Attractions of Idanre Hills, Ondo State, Nigeria. Am. J. Tour. Manag..

[22] Choo H., Ahn K., Petrick J.F. (2016). An integrated model of festival revisit intentions. Theory of planned behavior and festival quality/satisfaction. Int. J. Contemp. Hosp. Manag..

[23] Ritchie J.R.B., Crouch G.I. (2003). The Competitive Destination: A Sustainable Tourism Perspective.

[24] Chen C.C., Huang W.J., Petrick J.F. (2016). Holiday recovery experiences, tourism satisfaction and life satisfaction—Is there a relationship?. Tour. Manag..

[25] Le C.C., Dong D.X. (2017). Factors affecting european tourists’ satisfaction in Nha Trang city: Perceptions of destination quality. Int. J. Tour. Cities.

[26] Su W.-S., Chang L.-F., Yeh M.-T. (2017). Developing sustainable tourism attitude in Taiwanese residents. Int. J. Organ. Innov..

[27] Thao H.D.P., Thuy V.T.N., Dat P.M. (2019). Service quality attributes in ecotourism: The incorporation of experiencial effects. Int. J. Bus. Soc..

[28] Anuwichanont J., Serirat S., Mechinda P., Archarungroj P. (2020). Examining tourists’ attitude towards the religious tourism in thailand. Rev. Integr. Bus. Econ. Res..

[29] Beerli A., Martin J.D. (2004). Factors influencing destination image. Ann. Tour. Res..

[30] Chee X., Yang W. (August 2011). Understanding Tourist Motivation and Behavioural Intention to Visit a New Chinese Beach Destination: A Case Study of Potential Swedish Tourists’ Intention to Travel to Nordic Village of Hainan Island, China. Master’s Thesis.

[31] Chang L.L., Backman K.F., Huang Y.C. (2014). Creative tourism: A preliminary examination of creative tourists’ motivation, experience, perceived value and revisit intention. Int. J. Cult. Tour. Hosp. Res..

[32] Liu C.-H.S., Horng J.-S., Chou S.-F. (2015). A critical evaluation of sustainable tourism from the integrated perspective: Conducting moderated-mediation analysis. Tour. Manag. Perspect..

[33] Huang S., Hsu C. (2009). Travel motivation: Linking theory to practice. Int. J. Cult. Tour. Hosp. Res..

[34] Park D.B., Yoon Y.S. (2009). Segmentation by motivation in rural tourism: A Korean case study. Tour. Manag..

[35] Ballantyne R., Packer J., Falk J. (2011). Visitors’ learning for environmental sustainability: Testing short-and long-term impacts of wildlife tourism experiences using structural equation modelling. Tour. Manag..

[36] Zhang H., Lei S.L. (2012). A structural model of residents’ intention to participate in ecotourism: The case of a wetland community. Tour. Manag..

[37] Huang Y., Liu C.S. (2017). Moderating and mediating roles of environmental concern and ecotourism experience for revisit intention. Int. J. Contemp. Hosp. Manag..

[38] Murphy P.E. (1985). Tourism: A Community Approach.

[39] Gunn C.A. (1988). Tourism Planning.

[40] Gee C.Y., Mackens J.C., Choy. D.J. (1989). The Travel Industry.

[41] Gursoy D., Jurowsky C., Uysal M. (2002). Resident attitudes: A Structural Modeling Approach. Ann. Tour. Res..

[42] Vargas A., Plaza M., Porras N. Desarrollo del turismo y percepción de la comunidad local: Factores determinantes de su actitud hacia un mayor desarrollo turístico. Proceedings of the XXI Congreso Anual AEDEM, Universidad Rey Juan Carlos.

[43] Nunkoo R., So K.K.F. (2015). Residents’ support for tourism: Testing alternative structural models. J. Travel Res..

[44] Andereck K.L., Voght C.A. (2000). The relationship between residents’ attitudes toward tourism and tourism development options. J. Travel Res..

[45] Byrd E., Cardenan D., Dregalla S. (2009). Differences in stakeholder attitudes of tourism development and the natural environment. E-Rev. Tour. Res..

[46] Ekanayake E., Long A.E. (2012). Tourism development and economic growth in developing countries. Int. J. Bus. Financ. Res..

[47] Hutchinson J., Lai F., Wang Y. (2009). Understanding the relationships of quality, value, equity, satisfaction, and behavioral intentions among golf travelers. Tour. Manag..

[48] Castillo Canalejo A.M., Jimber Del Río J.A. (2018). Quality, satisfaction and loyalty índices. J. Place Manag. Dev..

[49] Kao Y.F., Huang L.S., Wu C.H. (2008). Effects of theatrical elements on experiential quality and loyalty intentions for theme parks. Asia Pac. J. Tour. Res..

[50] Chang N.J., Fong C.M. (2010). Green product quality, green corporate image, green customer satisfaction, and green customer loyalty. Afr. J. Bus. Manag..

[51] Wu H.-C., Ai C.-H., Cheng C.-C. (2016). Synthesizing the effects of green experiential quality, green equity, green image and green experiential satisfaction on green switching intention. Int. J. Contemp. Hosp. Manag..

[52] Roldán J., Sánchez-Franco M. (2012). Variance-Based Structural Equation Modeling: Guidelines for Using Partial Least Squares in Information Systems Research. Research Methodologies, Innovations and Philosophies in Software Systems Engineering and Information Systems.

[53] Sarstedt M., Hair J.F., Ringle C.M., Thiele K.O., Gudergan S.P. (2016). Estimation issues with PLS and CBSEM: Where the bias lies!. J. Bus. Res..

[54] Podsakoff P.M., MacKenzie S.B., Lee J.-Y., Podsakoff N.P. (2003). Common method biases in behavioral research: A critical review of the literature and recommended remedies. J. Appl. Psychol..

[55] Huber G.P., Power D.J. (1985). Retrospective reports of strategic-level managers: Guidelines for increasing their accuracy. Strateg. Manag. J..

[56] Harman H. Modern factor analysis. University of Chicago Press: Chicago, IL, USA, 1967.

[57] Podsakoff P.M., Organ D.W. (1986). Self-Report in Organizational Research: Problems and prospects. J. Manag..

[58] Carmines E.G., Zeller R.A. (1979). Reliability and Validity Assessment.

[59] Hair J.F., Hult G.T.M., Ringle C., Sarstedt M. (2017). A Primer on Partial Least Squares Structural Equation Modeling (PLS-SEM).

[60] Werts C.E., Linn R.L., Joreskog K.G. (1974). Interclass reliability estimates: Testing structural assumptions. Educ. Psychol. Meas..

[61] Nunnally J.C. (1978). Psychometric Theory.

[62] Fornell C., Larcker D.F. (1981). Evaluating structural equation models with unobservable variables and measurement error. J. Mark. Res..

[63] Carrión C.C., Nitzl C., Roldán J.L. (2017). Mediation Analyses in Partial Least Squares Structural Equation Modeling: Guidelines and Empirical Examples. Partial Least Squares Path Modeling.

[64] European Commission COVID-19: EU Guidance for the Progressive Resumption of Tourism Services and for Health Protocols in Hospitality Establishments, Brussels. 2020, May, 13. 3251. https://ec.europa.eu/info/sites/info/files/communication_tourismservices_healthprotocols.pdf.

[65] World Health Organization (WHO). https://www.who.int/publicationsdetail/water-sanitation-hygiene-and-waste-management-for-the-covid-19-virusinterim-guidance.

[66] Salathé M., Althaus C.L., Neher R., Stringhini S., Hodcroft E., Fellay J., Zwahlen M., Senti G., Battegay M., Wilder-Smith A. (2020). COVID-19 Epidemic in Switzerland: On the importance of testing, contact tracing and isolation. Swiss Med. Wkly..

[67] Weber M., Podnar Žarko I. (2019). Regulatory view on smart city services. Sensors.

[68] Dalton C.B., Corbett S.J., Katelaris A.L. (2020). Pre-emptive low cost social distancing and enhanced hygiene implemented before local COVID19 transmission could decrease the number and severity of cases. Med. J. Aust..

[69] Dinarto D., Wanto A., Sebastian L.C. Global Health Security—COVID-19: Impact on Bintan’s Tourism Sector. https://dr.ntu.edu.sg/bitstream/10356/137356/2/CO20033.pdf.

